# Editorial on Special Issue “Advances in Hydrogels”

**DOI:** 10.3390/gels8120787

**Published:** 2022-11-30

**Authors:** Yang Liu

**Affiliations:** 1 School of Pharmacy, Hengyang Medical School, University of South China, Hengyang 421001, China; liuyanghxl@126.com; Tel.: +86-0734-8281296; 2 Hunan Provincial Key Laboratory of Tumor Microenvironment Responsive Drug Research, Hengyang 421001, China; 3 Hunan Province Cooperative Innovation Center for Molecular Target New Drug Study, Hengyang 421001, China

Hydrogels are a class of soft materials with crosslinked network structures. They show good biocompatibility, biodegradability, hydrophilicity, and mechanical properties similar to those of tissue, so they have a wide range of applications. In recent years, a variety of multifunctional hydrogels with excellent performance have been developed, greatly expanding the depth and breadth of their applications. This Special Issue focuses on the recent advances regarding hydrogels, aiming to provide reference for researchers in related fields. We have collected thirteen original research articles and three valuable reviews from thirteen different countries including Canada, China, Thailand, Mexico, India, Saudi Arabia, Chile, Germany, the Czech Republic, Colombia, Romania, Israel, and the USA.

Hydrogels can be prepared through different crosslinking methods. Photo-crosslinking has attracted much attention due to its advantages of mild preparation conditions, and convenient and simple operation. Liu and colleagues summarized the types of photo-crosslinked hydrogel monomers, the methods for the preparation of photo-crosslinked hydrogels with different morphologies, and their applications in biomedical engineering [1]. Gradilla-Orozco et al. prepared a multi-structured poly(potassium acrylate-co-acrylamide)-based hydrogel with a fractal-like structure via photo-crosslinking [2]. Sabel-Grau and co-workers developed a poly(ethylene glycol)-diacrylate (PEG-DA)-based photo-crosslinked hydrogel [3]. They used erythrosin B or eosin Y as the novel photoinitiator, which increased the multifunctionality of the prepared hydrogels. Different crosslinking methods and crosslinking degrees affect the properties of obtained hydrogels. To study the effect of the crosslinking degree on the adhesivity of hydrogels, Li et al. prepared polyvinyl alcohol (PVA), polyacrylamide (PAM) and polyvinyl alcohol-bearing styrylpyridinium group (PVA-SbQ) hydrogels through freeze–thaw cycles, thermal crosslinking and photo-crosslinking, respectively [4]. They found that the adhesion capability of these hydrogels decreased with an increase in the crosslinking degree. However, the adhesion could be improved by maintaining the adhesive functional groups and enhancing the flexibility of the polymer chains. Moreover, the synthesis of responsive hydrogels with excellent injectability and biodegradability is also attractive. Steinman and Domb synthesized a sensitive poly(ethylene glycol)-b-poly(lactic acid)-S-S-poly(lactic acid)-b-poly(ethylene glycol) (PEG-PLA-SS-PLA-PEG) copolymer by the grafting of PEG via urethane linkages, which could form a hydrogel above 32 °C, collapse immediately upon the reaction with NaBH_4_, and be degraded slowly by hydrolytic degradation [5]. This hydrogel may be applied in drug-delivery vehicles with slow-release behavior and immediate-release behavior under the action of reducing agents.

According to the source, hydrogels can be divided into natural and synthetic hydrogels. Natural hydrogels generally have many potential sources, are low cost, and may show better biocompatibility, biodegradability, and other properties, which has attracted much attention. Heger and colleagues investigated the influence of a natural phospholipid lecithin (L-α-phosphatidylcholine) on three differently crosslinked hydrogels (physically crosslinked agarose, ionically crosslinked alginate, and a chemically crosslinked mixture of PVA and chitosan) [6]. They found that lecithin could modify the internal architecture and mechanical properties of hydrogels. Múnera-Tangarife and co-workers prepared a natural carboxymethyl cellulose (CMC)-based transparent film with good barrier properties preventing the passage of oxygen and fats, and studied the factors affecting the drying process for CMC using refractance window-conductive hydro-drying (RW-CHD) [7]. Călina et al. also synthesized a series of superabsorbent hybrid hydrogel compositions constructed from natural xanthan gum (XG)/CMC/graphene oxide (GO) by e-beam radiation crosslinking [8].

With the deepening of research, various multifunctional hydrogels with excellent performance have played an increasingly important role in biomedical fields such as tumor treatment, cell scaffolds, wound repair, biological detection, the food industry and controlled drug release. Wang et al. developed a simple and powerful alginate–Ca^2+^ hydrogel (ACH) to load commercial copper sulfide (CuS) powders for local tumor NIR-II photothermal therapy (PTT) [9]. This hydrogel exhibited a good photothermal capacity and stability. Under 1064 nm NIR-II laser irradiation for 5 min, the temperature enhancement of hydrogels with different concentrations of CuS increased from 17.3 °C to 38.1 °C. These hydrogels had low cytotoxicity toward 4T1 cells, but the toxicity increased in a CuS-concentration- and laser-power-density-dependent manner under radiation. Animal experiments also showed that the large CuS particles in the hydrogel could very efficiently accumulate in tumor tissues and clearly suppress the tumors without causing evident inflammatory lesions or organ damage. Wangsawangrung and co-workers developed a quercetin/hydroxypropyl-β-cyclodextrin (HP-β-CD) inclusion complex-loaded polyvinyl alcohol (PVA) hydrogel that was physically crosslinked through multiple freeze–thaw cycles [10]. The quercetin/HP-β-CD inclusion complex was prepared via the solvent evaporation method, which showed significant antioxidant activity compared with free quercetin. An MTT assay also showed that the viability of mouse fibroblast NCTC 929 clone cells cultured with various concentrations of extracted media from these hydrogels was greater than 70%, indicating that these hydrogels showed low cytotoxicity. All the results illustrate that these hydrogels with the quercetin/HP-β-CD inclusion complex were attractive candidates for wound healing. We also constructed thermosensitive hydrogel scaffolds for cell culture [11]. These hydrogels were formed through the thermally induced gelation of thermosensitive microgels, which were prepared by the radical polymerization of 2-methyl-2-propenoic acid-2-(2-methoxyethoxy) ethyl ester (MEO_2_MA) and oligoethylene glycol methyl ether methacrylate (OEGMA). The prepared thermosensitive hydrogels could be used for the in situ embedding and three-dimensional (3D) culture of MCF-7 breast cancer cells. The cells grew rapidly in the 3D scaffold and maintained a high proliferative capacity.

Molecularly imprinted polymeric hydrogel (MIPG)-based fluorescent sensors for commercial applications were developed by Zou and colleagues [12]. The MIPG for the detection of zearalenone (ZON) (MIPG_ZON) was first prepared using 4-vinylpyridine (4-VPY) as the functional monomer and ethylene glycol dimethacrylate (EGDMA) as the crosslinker for the ZON. This MIPG_ZON optical sensor showed excellent stability and reproducibility, and could detect ZON in commercial corn juice in a linear fashion across the concentration range 0–10 μM, with a limit of detection (LOD) of 1.6 μM. In parallel, a MIP-based fluorescent probe for the detection of glucuronic acid was also fabricated for cell imaging. Casas-Forero et al. prepared four types of commercial confectionary hydrogels through the introduction of a cryoconcentrated blueberry juice (CBJ) rich in polyphenols, anthocyanins, and flavonoids into a gelatin gel (GG), aerated gelatin gel (AGG), gummy (GM), and aerated gummy (AGM), respectively [13]. The structures of the hydrogels could protect the CBJ during in vitro digestion, which enhanced the bioaccessibility of the CBJ. Ahmed and colleagues also developed a type of nanogel matrix soft confectionary for the oral supplementation of vitamin D, which resulted in the enhancement of bioavailability, stability, and patient compliance [14]. Moreover, Sahan et al. highlighted the biomedical applications and future perspectives of biomimetic hydrogels in the study of cancer mechanobiology [15]. Additionally, novel hydrogels with new formulations for the topical administration of therapeutic agents were also reviewed by Almoshari [16].

The articles and reviews presented in this Special Issue offer a real insight into the advances regarding hydrogels. It is very clear that many novel multifunctional hydrogels will continue to emerge with bright application prospects from the efforts of researchers.
